# Insulin Resistance in relation to Hypertension and Dyslipidaemia among Men Clinically Diagnosed with Type 2 Diabetes

**DOI:** 10.1155/2023/8873226

**Published:** 2023-05-26

**Authors:** Huseini Alidu, Peter Paul Mwinsanga Dapare, Lawrence Quaye, Nafiu Amidu, Simon Bannison Bani, Moses Banyeh

**Affiliations:** ^1^Department of Medical Laboratory Science, University of Health and Allied Sciences, Ho, Ghana; ^2^Department of Biomedical Laboratory Science, University for Development Studies, Tamale, Ghana

## Abstract

Pathophysiologically, type 2 diabetes can result from insulin resistance or insulin insufficiency alone. It is unclear whether relative insulin shortage or pronounced insulin resistance is linked to poor cardiometabolic problems like obesity. Therefore, the objective of this study was to evaluate the relationship between insulin resistance (IR), hypertension, and dyslipidaemia, in men with type 2 diabetes mellitus. One hundred and twenty-one (121) type 2 diabetic men participated in this cross-sectional study, which was conducted between September 2018 and September 2019. Sociodemographic information was collected using a self-designed questionnaire. Anthropometric data were also taken and blood samples collected for estimation of insulin, glucose, and lipid concentrations. HOMA-IR was calculated from the fasting insulin and glucose values, and a HOMA − IR ≥ 2 was considered to indicate insulin resistance. Of the 121 participants, 39.7% were classified as insulin-resistant. Levels of total cholesterol (4.82 ± 1.2 mmol/L; *p* = 0.007 vs. 4.25 ± 1.1 mmol/L), LDL cholesterol (3.17 ± 0.9 mmol/L; *p* = 0.001 vs. 2.52 ± 0.8 mmol/L), and TC/HDL-C ratio (3.93 ± 0.9; *p* = 0.042 vs. 3.58 ± 0.9) and the prevalence of abnormal LDL-C (14.6%; *p* = 0.015 vs. 2.7%) and elevated BP (83.3%; *p* = 0.048 vs. 67.1%) were higher in the insulin-resistant group. LDL cholesterol (AUC = 0.670; *p* = 0.001) better classified subjects as being insulin-resistant compared to other lipid markers. The odds of insulin resistance in dyslipidaemia were not statistically significant after adjusting for obesity. The link between insulin resistance and dyslipidaemia and hypertension in male diabetics may thus be mediated by obesity.

## 1. Introduction

T2DM accounts for more than 90% of diabetes cases. T2DM is characterized by the existence of tissue insulin resistance (IR), a deficit in insulin secretion, and a lack of compensatory responses to insulin secretion [1, 2]. Type 2 diabetes mellitus (T2DM) is a chronic condition characterized by insulin resistance and beta-cell dysfunction. Insulin resistance refers to the inability of cells to effectively use insulin, leading to high levels of insulin (hyperinsulinemia) in the body. Beta-cell dysfunction refers to a relative deficiency in insulin production by the pancreas. It is currently not clear to what extent each of these factors contributes to the development of T2DM [3–5]. Historically, insulin resistance has been understood primarily in terms of its impact on glucose metabolism, but it is now known that insulin also plays a role in a variety of other processes such as lipid and protein metabolism, ion and amino acid transport, cell cycle and differentiation, and NO synthesis [6].

In the vascular system, insulin typically promotes vasodilation through the production of NO. However, in the case of insulin resistance, the impaired synthesis of NO and impaired vasodilation may contribute to increased blood pressure (BP) [7]. Insulin resistance has been linked to several mechanisms that may increase BP, including enhanced tissue angiotensin II and aldosterone activity, increased sympathetic nervous system activity, and oxidative stress [8–10]. Insulin resistance has been suggested to potentially play a role in the development of essential hypertension. The relationship between insulin resistance and essential hypertension may be causal, meaning that insulin resistance causes essential hypertension, or it may be a noncausal association, meaning that the two variables are correlated but not necessarily directly related [11].

Diabetic dyslipidaemia, characterized by high levels of triglycerides and low levels of high-density lipoprotein cholesterol (HDL-C), is thought to contribute to the development of atherosclerosis in individuals with diabetes mellitus [12, 13]. A number of studies have found a strong link between high levels of fasting triglycerides and an increased risk of coronary artery disease (CAD) in people with diabetes [13–15]. Abnormalities in lipid metabolism have been identified as a key factor in the development of insulin resistance [16]. People with insulin resistance often have a distinctive lipid profile, including decreased levels of HDL cholesterol, increased levels of VLDL cholesterol, and, less commonly, increased levels of LDL cholesterol. The synthesis of VLDL is regulated by the concentration of insulin in the blood and the availability of nutrients. There is evidence to suggest that insulin resistance, which is often accompanied by hyperinsulinemia, leads to increased VLDL synthesis by the liver and contributes to elevated triglyceride levels in people with type 2 diabetes [17].

Type 2 diabetes mellitus is typically diagnosed based on the presence of elevated blood glucose levels that can be managed without the use of exogenous insulin [18]. Many individuals with type 2 diabetes may have some degree of insulin resistance and may also have a relative deficiency in insulin production by the pancreas. The specific balance of insulin resistance and insulin deficiency may vary from person to person and may also change over time. It is not clear which of these factors is most important in the development of type 2 diabetes in an individual patient, and clinical decisions about treatment often involve a combination of approaches targeting both insulin resistance and insulin deficiency [19].

It is not yet clear whether individuals with type 2 diabetes who are more insulin-resistant or who have a relative deficiency in insulin production are more closely associated with hypertension and dyslipidaemia. The relationship between insulin resistance, hypertension, and dyslipidaemia in type 2 diabetes in men is complex and requires further investigation. Improved understanding of the association and effective interventions targeting this relationship could potentially improve the health and well-being of this population and reduce the burden of type 2 diabetes. Additionally, studies which include both men and women may lack statistical power to detect meaningful results for men. This study therefore sought to assess the relationship between insulin resistance, hypertension, and dyslipidaemia among men clinically diagnosed with type 2 diabetes.

## 2. Materials and Methods

### 2.1. Study Population and Design

This cross-sectional study was conducted among male diabetic subjects from September 2018 to September 2019. Adult male type 2 diabetics, visiting a selected tertiary health facility in Ghana, were selected using a convenience sampling technique. Type 1 diabetics and morbid type 2 diabetic were excluded from the study.

#### 2.1.1. Sample Size

On the assumption that 6.5% of the general population is diabetic, the minimum sample size for this study was estimated to be 93 adult male diabetics [20]. A type 1 error (*α*) of 0.05 and an anticipated difference between the sample and the overall population of 5% were considered.

(1) $$\begin{array}{c} n=\frac{z^{2}p\left(1-p\right)}{d^{2}}, \end{array}$$

where *n* is the minimal sample size, *d* is the absolute standard error = 0.05, *p* is the prevalence = 6.5%, and *z* is the standard normal variance, 1.96, a power of 95% CI (*β* = 5%) and 5% type 1 error probability.

The sample size was recalculated in this study, restricted to only adult male diabetics who responded to not less than 80% of the questionnaire items, to account for any potential loss of respondents. The sample size was computed with a response rate of 80% as follows: 93/0.80. The estimated sample size was roughly 116 using the formula above. One hundred and twenty-one (121) individuals were therefore selected for this study.

### 2.2. Data Collection

#### 2.2.1. Anthropometric and Sociodemographic Data

Participants in the study who provided their assent were given a self-designed semistructured questionnaire to fill out regarding sociodemographic data like age, marital status, highest educational level, smoking, occupation, alcohol consumption, level of exercise, income, and duration of diabetes. Any action lasting more than 30 minutes that caused light sweating, a slight to moderate increase in respiration, or an increase in heart rate was considered exercise. Whether a person had the habit of smoking at least one cigarette each day determined whether they were considered a smoker. Education was divided into three levels: basic, secondary, and tertiary.

A Gulick II spring-loaded measuring tape was used to measure the waist circumference (to the closest centimeter) halfway between the suprailiac crest and the inferior angle of the ribs (Gay Mills, WI, USA). Hip circumference was estimated using the largest circumference over the buttocks. Waist to hip ratio was estimated as waist circumference (cm) divided by hip circumference (cm). A WHR of ≥0.90 was considered obese.

#### 2.2.2. Blood Pressure

Using a sphygmomanometer cuff and a stethoscope, blood pressure was taken while the subject was seated at the level of the heart. According to the American Heart Association's recommendation, measurements were collected from the left brachial artery after patients had been seated for at least five (5) minutes [21]. The diastolic value was calculated using the fifth Korotkoff sound, phase V (lack of sound), rather than phase IV (muffling). The mean reading was recorded to the closest 2.0 mmHg in triplicate measurements with a five (5)-minute rest period in between observations. A blood pressure of 140/90 mmHg was considered hypertensive.

#### 2.2.3. Sample Collection, Preparation, and Analysis

Following an overnight fast, each subject had ten milliliters of venous blood taken from them into a fluoride oxalate tube and serum separator tube (SST) under strict aseptic conditions (Becton Dickinson, Rutherford, NJ). When it came the time for the biochemical investigations, samples in SSTs were centrifuged at 3000 g for 5 minutes, and the serum was aliquoted and kept in cryovials at a temperature of -70°C. On the other hand, samples in fluoride oxalate tubes were used for fasting blood glucose measurement. The Mindray BS-240 Chemistry Analyzer (Mindray, China) was used to measure lipids and fasting blood glucose levels. MedSource Diagnostics reagents were utilized in each of these assays. The AxSYM analyzer was used to conduct the hormonal assays. Microparticle enzyme immunoassay is used by the AxSYM to measure the levels of adiponectin, leptin, and insulin. All hormonal assays were performed using the Elabscience® reagent kit. The automated equipment used procedures that followed the reagent makers' directions to determine biochemical and hormonal parameters.

### 2.3. Definition of Terms

#### 2.3.1. Homeostatic Model Assessment for Insulin Resistance (HOMA-IR)

HOMR-IR was estimated based on the following mathematical formula: (fasting insulin (mlU/L) × fasting glucose (mmol/L))/22.5 [22]. Values ≥ 2 were considered to indicate insulin resistance [23].

#### 2.3.2. Quantitative Insulin Sensitivity Check Index (QUICKI)

QUICKI was calculated using the following mathematical formula: 1/(log (fasting insulin (mU/L)) + log (fasting blood sugar (mg/dL))). Lower values indicate greater insulin resistance, and values below 0.34 were considered to indicate insulin resistance [24].

#### 2.3.3. Homeostatic Model Assessment of *β*-Cell Function (HOMA-B)

HOMA-B was calculated based on the following formula: 20 × fasting insulin (mU/L)/fasting glucose (mmol/mL) − 3.5 [22].

### 2.4. Statistical Analysis

Analyses were conducted using MedCalc® version 10.2.0.0 (http://www.medcalc.be), GraphPad® version 9.0 (San Diego, California, USA), and the Statistical Package for Social Sciences (SPSS) version 26 (Armonk, NY: IBM Corp). Indicators of data included mean, standard deviation, and percentages. When comparing continuous variables, the unpaired *T*-test and Mann-Whitney tests were used, and when comparing categorical variables, the chi-square test was used. The correlation between the variables was assessed using Pearson's correlation. Receiver operating characteristics (ROC) were used to evaluate the relative propensities of various characteristics to predict the dependent variable. All statistical analyses were considered significant at a value of <0.05.

## 3. Results

One hundred and thirty (130) questionnaires were administered in total, of which 125 (96%) followed through to the end of the data collection. 121 completed and evaluable questions, representing a response rate of 93%, were left after the set of questionnaires from 4 people was rejected because they were insufficiently completed. Following stratification based on a HOMA − IR ≥ 2, forty-eight (48) participants representing 39.7% had insulin resistance.

### 3.1. Sociodemographic Characteristics of Study Population Stratified by HOMA-IR

Table 1 shows the general and sociodemographic characteristics of the studied population. The average age of the study population was 63.0 ± 10.8 years with the majority (95%) of them being married. About half (48%) of the participants had attained at least secondary education.

When the population was stratified based on HOMA-IR, the proportion of subjects with at least secondary education (60%) was significantly higher in the HOMA − IR < 2 group than in their counterparts with a HOMA − IR ≥ 2 (Table 1).

### 3.2. Biochemical and Haemodynamic Profile of Study Population

Table 2 summarises the biochemical and haemodynamic profile of the study population stratified by HOMA-IR. The mean FBG, insulin, and HOMA-IR were 9.0 ± 2.7 mmol/L, 114.1 ± 174.7 pg/mL, and 1.5 ± 2.3, respectively. The mean WHR was 0.93 ± 0.04. The average SBP and DBP were 158.1 ± 25.8 mmHg and 100.9 ± 13.5 mmHg, respectively. The mean total cholesterol, triglyceride, HDL cholesterol, and LDL cholesterol were 4.47 ± 1.2 mmol/L, 0.9 ± 0.4 mmol/L, 1.27 ± 0.4 mmol/L, and 2.78 ± 0.9 mmol/L, respectively. The TC/HDL ratio and TG/HDL ratio of the studied population were 3.72 ± 0.9 and 1.76 ± 0.9, respectively.

When the population was stratified based on their HOMA-IR, FBG, insulin levels, HOMA-IR, and HOMA-B were significantly higher among subjects with HOMA − IR ≥ 2 compared to their counterparts. However, QUICKI was significantly lower in the HOMA − IR ≥ 2 subjects compared to the HOMA − IR < 2 group. Also, WHR, total cholesterol, LDL cholesterol, and TC/HDL ratio were higher among the HOMA − IR ≥ 2 (0.95 ± 0.04, *p* < 0.0001; 4.82 ± 1.2 mmol/L, *p* = 0.007; 3.17 ± 0.9 mmol/L, *p* = 0.001; and 3.93 ± 0.9, *p* = 0.042, respectively) group compared to their counterparts with HOMA − IR < 2 (0.92 ± 0.04, 4.25 ± 1.1 mmol/L, 2.52 ± 0.8 mmol/L, and 3.58 ± 0.9) as shown in Table 2.

### 3.3. Prevalence of Hypertension and Dyslipidaemia

Table 3 shows the proportions of the study population with elevated blood pressure and abnormal lipid parameters. Most (73.6%) of the studied participants had elevated blood pressure, most (78.5%) of whom indicated having hypertension on diagnosis of their diabetes. Majority of the population had normal total cholesterol (71%), triglyceride (91%), HDL cholesterol (68%), and LDL cholesterol (93%). However, more than half (63%) had at least one abnormal lipid (dyslipidaemia) parameter.

When the population was stratified based on HOMA-IR, the proportions of subjects with elevated blood pressure and high LDL cholesterol were significantly higher in the HOMA − IR ≥ 2 (83.3%, *p* = 0.048 and 14.6%, *p* = 0.015) group compared to their counterparts with HOMA − IR < 2 (67.1% and 2.7%, respectively), as shown in Table 3.

### 3.4. Haemodynamic and Lipid Determinants of Insulin Resistance

The impact of haemodynamic and lipid parameters on the occurrence of insulin resistance is shown in Table 4. A high LDL cholesterol (OR = 6.1, *p* = 0.029) was found to significantly affect the occurrence of insulin resistance (HOMA − IR ≥ 2). After adjusting for obesity (WHR), none of the variables could independently predict the occurrence of insulin resistance as shown in Table 4.

### 3.5. Correlation of Insulin and Indices of Insulin Resistance against Haemodynamic and Lipid Parameters

Pearson's correlation was done to assess the association between insulin, insulin resistance indices (HOMA-IR and QUICKI), index of pancreatic beta function (HOMA-B), and haemodynamic and lipid parameters as shown in Table 5. HOMA-IR showed significant positive correlations with atherogenic index of plasma (*r* = 0.18, *p* < 0.05). QUICKI showed significant inverse relations with systolic blood pressure (*r* = −0.20, *p* < 0.05), total cholesterol (*r* = −0.18, *p* < 0.05), and LDL cholesterol (*r* = −0.25, *p* < 0.01) as shown in Table 5.

### 3.6. Receiver Operating Characteristics (ROC) for Haemodynamic and Lipid Parameters

**Figure 1:**
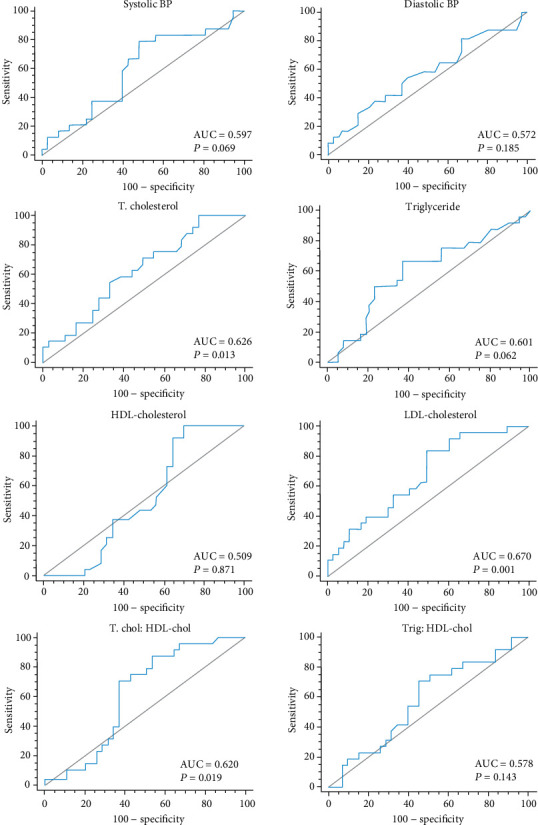
ROC curves for blood pressure and lipid parameters in classifying insulin resistance.

The ROC curves and the area under curve (AUC) showing the comparable abilities of haemodynamic and lipid parameters to classify insulin resistance are shown in Figure 1, and their respective cut-off points for classification of insulin resistance are shown in Table 6. Total cholesterol (AUC = 0.626, *p* = 0.013), LDL cholesterol (AUC = 0.670, *p* = 0.001), and TC/HDL (AUC = 0.620, *p* = 0.019) significantly classified subjects as insulin-resistant, with LDL cholesterol showing the highest AUC for the classification of insulin resistance.

A higher Youden index indicates higher diagnostic value as evaluated by a balance between the sensitivity and specificity of a marker. From Table 6, LDL cholesterol (0.34) and TC/HDL ratio (0.34) showed the highest Youden indices at diagnostic cut-offs of >2.3 mmol/L (sensitivity = 83.3 and specificity = 50.7) and >3.12 (sensitivity = 87.5 and specificity = 46.6), respectively.

## 4. Discussion

The study assessed the link between insulin resistance, hypertension, and dyslipidaemia and observed a 73.6% prevalence of elevated blood pressure among the study population. This figure is slightly lower than the self-reported figure of 78.5% by the study participants. The prevalence of hypertension in this study is similar to the 73.7% among type 2 diabetic men reported by Hu et al. [25]. It is however higher than the 66.8% reported by Vasanthakumar and Kambar [26], 62.1% reported by Mogre et al. [27], and 65% by Akalu and Belsti [28]. The contrasting figures between this study and those of the other studies could be attributed to the differences in cut-offs used, levels of urbanisation, and socioeconomic status which are known to significantly affect the prevalence of hypertension among different populations.

The prevalence of hypertension was higher among the insulin-resistant group, and systolic blood pressure showed significant inverse correlation with insulin sensitivity. The association between hypertension and insulin resistance has seen conflicting findings from different studies. In this study, even though the frequency of elevated blood pressure was significantly higher among the insulin-resistant group, it was not independently associated with insulin resistance after adjusting for obesity and other confounding variables. Ferrannini et al. [29] demonstrated a link between insulin resistance and essential hypertension in a general population. It is unclear how insulin resistance is connected to high blood pressure and hypertension. It is believed that compensatory hyperinsulinemia brought on by insulin resistance could result in hypertension. Hypertension may be caused by insulin's stimulation of the sympathetic nervous system, increase in salt retention in the kidneys, modulation of cation transport, and induction of vascular smooth muscle hypertrophy. In consonance with the findings in this study, however, Saad et al. [30] found no relation between insulin resistance and hyperinsulinemia and hypertension among diabetics, after adjusting for age, obesity, and sex. They explained that acute insulin infusion has a vasodilator hypotensive effect and that insulin could lower blood pressure in diabetics [31, 32]. They however add that the possibility of resistance to the vasodilator effect of insulin in an insulin-resistant state could lead to a rise in blood pressure. Consequently, the association between insulin resistance and hypertension may not be causal but rather the two are linked indirectly through other metabolic abnormalities such as obesity, as observed in this study.

Lipid profile analysis of the male diabetic subjects showed 62.8% subjects with at least one lipid abnormality. Diabetes has been shown to be an independent risk factor for the development of dyslipidaemia, and the phenomenon is well recognised [33, 34]. The prevalence in this study is compared to the 61.3% among diabetic men in a study by Haile and Timerga [35] but higher than the 43.3% reported by Li et al. [36] and lower than the 94% and 72.6% reported by Omodanisi et al. [37] and Ahmmed et al. [38], respectively.

Relations between different lipid parameters and insulin resistance have been studied in the past [39–41], and it is quite clear that there is an association between dyslipidaemia and insulin resistance. The levels of total cholesterol (TC), LDL cholesterol, and total cholesterol to HDL cholesterol ratio (TC/HDL) were significantly higher in the insulin-resistant group. Similarly, TC, LDL cholesterol, and TC/HDL showed significant direct correlations with insulin resistance. Also, the prevalence of subjects with high LDL cholesterol was significantly higher among the insulin-resistant group. Based on in vitro studies, some researchers have hypothesized that excessive levels of triglyceride-rich VLDL particles could impair insulin action by preventing insulin from attaching to its receptor; i.e., insulin resistance might be a secondary symptom of primary dyslipidaemia [42, 43]. Other researchers have however failed to show this causal effect [44]. Garg et al. [45] showed that marked reduction in hypertriglyceridaemia did not lead to an improvement in the insulin sensitivity of patients with hypertriglyceridaemia, thus indicating that insulin resistance is probably the underlying mechanism of dyslipidaemia. Upon adjusting for obesity as a confounding factor, none of the lipid parameters was independently associated with insulin resistance in this study; hence, the association between increased LDL-C and insulin resistance may be mediated by obesity, which is known to cause dyslipidaemia independent of insulin resistance [46].

The comparative ability of various lipid markers as screening tools for insulin resistance was also explored in this study. LDL cholesterol concentration showed superiority in the classification of insulin resistance and at a cut-off of >2.3 mmol/L. LDL-C may prove useful in the classification of insulin resistance among diabetic men. Insulin resistance is characterized by increased LDL-C concentration, but the role and importance of LDL-C in the classification of IR is not fully investigated and is unclear from this current study.

## 5. Conclusion

Insulin resistance is associated with hypertension and dyslipidaemia among type 2 diabetic men. This may however be mediated by obesity associated with insulin resistance. An LDL-C cut-off of >2.3 mmol/L better classifies insulin resistance in type 2 diabetic men.

## Figures and Tables

**Table 1:** General and sociodemographic characteristics stratified by HOMA-IR.

Parameter	Total (*n* = 121)	HOMA − IR < 2 (*n* = 73)	HOMA − IR ≥ 2 (*n* = 48)	*p* value
Age (years)	63.0 ± 10.8	63.1 ± 11.48	62.8 ± 9.8	0.872^b^
Marital status				
Married	115 (95%)	69 (94.5%)	46 (95.8%)	0.745^a^
Single	6 (5%)	4 (5.5%)	2 (4.2%)	
Highest education				
Basic	63 (52%)	30 (41.1%)	33 (68.8%)	0.005^a^
Secondary	52 (43%)	37 (50.7%)	15 (31.3%)	
Tertiary	6 (5%)	6 (8.2%)	0 (0%)	
Exercise per week	1.8 ± 0.9	1.9 ± 0.9	1.7 ± 1.0	0.382^b^
Duration of diabetes (years)	8.3 ± 6.2	7.55 ± 6.1	9.5 ± 6.3	0.096^b^

Data is presented as proportions and mean ± SD. Categorical variables were compared using chi-square^a^ test and continuous variables using unpaired *T*-test^b^.

**Table 2:** Biochemical and haemodynamic profile of study population stratified by HOMA-IR.

Parameter	Total (*n* = 121)	HOMA − IR < 2 (*n* = 73)	HOMA − IR ≥ 2 (*n* = 48)	*p* value
Fasting blood glucose (mmol/L)	9 ± 2.7	8.0 ± 2.6	10.5 ± 2.2	<0.0001
Insulin (pg/mL)	114.1 ± 174.7	3.24 ± 7.6	282.6 ± 172.4	<0.0001
Insulin (mU/L)	3.31 ± 5.1	0.1 ± 0.2	8.2 ± 5.0	<0.0001
HOMA-IR	1.51 ± 2.34	0.03 ± 0.08	3.76 ± 2.32	<0.0001
QUICKI	0.82 ± 0.44	1.14 ± 0.24	0.32 ± 0.03	<0.0001
HOMA-B	11.24 ± 20.2	0.52 ± 0.93	27.54 ± 24.30	<0.0001
Fasting blood glucose (mmol/L)	9 ± 2.7	8.0 ± 2.6	10.5 ± 2.2	<0.0001
Waist to hip ratio	0.93 ± 0.0	0.92 ± 0.0	0.95 ± 0.0	<0.0001
Systolic blood pressure (mmHg)	158.10 ± 25.8	154.60 ± 25.4	163.30 ± 25.8	0.069
Diastolic blood pressure (mmHg)	100.90 ± 13.5	99.19 ± 12.2	103.60 ± 15.1	0.082
Pulse (beats/min)	84.71 ± 14.1	85.68 ± 13.4	83.23 ± 14.9	0.349
Total cholesterol (mmol/L)	4.47 ± 1.2	4.25 ± 1.1	4.82 ± 1.2	0.007
Triglyceride (mmol/L)	0.90 ± 0.4	0.86 ± 0.4	0.97 ± 0.4	0.158
HDL cholesterol (mmol/L)	1.27 ± 0.4	1.29 ± 0.5	1.26 ± 0.3	0.712
LDL cholesterol (mmol/L)	2.78 ± 0.9	2.52 ± 0.8	3.17 ± 0.9	0.001
Atherogenic index of plasma	0.19 ± 0.2	0.17 ± 0.2	0.22 ± 0.2	0.242
TC/HDL	3.72 ± 0.9	3.58 ± 0.9	3.93 ± 0.9	0.042
TG/HDL	1.76 ± 0.9	1.72 ± 0.9	1.83 ± 0.7	0.513

Data is presented as mean ± SD and analyzed using unpaired *T*-test.

**Table 3:** Distribution of elevated blood pressure and abnormal lipid parameters stratified by HOMA-IR.

Parameter	Total (*n* = 121)	HOMA − IR < 2 (*n* = 73)	HOMA − IR ≥ 2 (*n* = 48)	*p* value
Systolic blood pressure				
Normal	30 (24.8%)	22 (30.1%)	8 (16.7%)	0.093
Elevated	91 (75.2%)	51 (69.9%)	40 (83.3%)	
Diastolic blood pressure				
Normal	20 (16.5%)	14 (19.2%)	6 (12.5%)	0.333
Elevated	101 (83.5%)	59 (80.8%)	42 (87.5%)	
Blood pressure				
Normal	32 (26.4%)	24 (32.9%)	8 (16.7%)	0.048
Elevated	89 (73.6%)	49 (67.1%)	40 (83.3%)	
Hypertension on diagnosis				
No	26 (21.5%)	14 (19.2%)	12 (25%)	0.446
Yes	95 (78.5%)	59 (80.8%)	36 (75%)	
Total cholesterol				
Normal	86 (71.1%)	55 (75.3%)	31 (64.6%)	0.202
High	35 (28.9%)	18 (24.7%)	17 (35.4%)	
Triglyceride				
Normal	110 (90.9%)	67 (91.8%)	43 (89.6%)	0.681
High	11 (9.1%)	6 (8.2%)	5 (10.4%)	
HDL cholesterol				
Normal	82 (67.8%)	48 (65.8%)	34 (70.8%)	0.559
Low	39 (32.2%)	25 (34.2%)	14 (29.2%)	
LDL cholesterol				
Normal	112 (92.6%)	71 (97.3%)	41 (85.4%)	0.015
High	9 (7.4%)	2 (2.7%)	7 (14.6%)	
Dyslipidaemia				
No	45 (37.2%)	28 (38.4%)	17 (35.4%)	0.743
Yes	76 (62.8%)	45 (61.6%)	31 (64.6%)	
Atherogenic index				
Normal	62 (51.2%)	40 (54.8%)	22 (45.8%)	0.335
Abnormal	59 (48.8%)	33 (45.2%)	26 (54.2%)	

Data is presented as proportions and mean ± SD where appropriate. Categorical variables were compared using chi-square test and continuous variables using unpaired *T*-test.

**Table 4:** Effects of blood pressure and lipid parameters on insulin resistance.

Parameter	OR (95% CI)	*p* value	aOR (95% CI)	*p* value
Waist to hip ratio				
Normal	1.0	—	1.0	—
Obese	3.3 (1.51-7.15)	0.003	9.1 (2.15-38.03)	0.003
Systolic blood pressure				
Normal	1.0	—	1.0	—
Elevated	2.2 (0.87-5.35)	0.097	0.9 (0.24-3.05)	0.818
Diastolic blood pressure				
Normal	1.0	—	1.0	—
Elevated	1.7 (0.59-4.68)	0.337	1.1 (0.26-4.4)	0.930
Blood pressure				
Normal	1.0	—	1.0	—
Elevated	2.5 (0.99-6.04)	0.052	0.7 (0.22-2.52)	0.631
Hypertension on diagnosis				
No	1.0	—	1.0	—
Yes	0.7 (0.3-1.71)	0.447	0.3 (0.09-1.26)	0.106
Total cholesterol				
Normal	1.0	—	1.0	—
High	1.7 (0.76-3.71)	0.204	1.1 (0.42-2.85)	0.844
Triglyceride				
Normal	1.0	—	1.0	—
High	1.3 (0.37-4.52)	0.681	0.9 (0.22-3.49)	0.852
HDL cholesterol				
Normal	1.0	—	1.0	—
Low	0.8 (0.36-1.74)	0.559	0.9 (0.42-2.29)	0.956
LDL cholesterol				
Normal	1.0	—	1.0	—
High	6.1 (1.2-30.56)	0.029	5.8 (0.96-35.03)	0.056
Dyslipidaemia				
No	1.0	—	1.0	—
Yes	1.1 (0.53-2.42)	0.744	1.1 (0.50-2.40)	0.831

**Table 5:** Partial correlation of indices of insulin resistance against blood pressure and lipid parameters.

Parameter	Insulin	HOMA-IR	QUICKI	HOMA-B
	*r*	*r*	*r*	*r*
Systolic blood pressure (mmHg)	0.16	0.14	-0.20^∗^	0.14
Diastolic blood pressure (mmHg)	0.17	0.14	-0.17	0.17
Pulse (beats/min)	-0.07	-0.07	0.21	-0.05
Total cholesterol (mmol/L)	0.12	0.10	-0.18^∗^	0.11
Triglyceride (mmol/L)	0.14	0.15	-0.15	0.12
HDL cholesterol (mmol/L)	-0.07	-0.08	0.02	-0.06
LDL cholesterol (mmol/L)	0.17	0.15	-0.25^∗∗^	0.15
Atherogenic index of plasma	0.17	0.18^∗^	-0.14	0.16
TC/HDL	0.15	0.15	-0.14	0.14
TG/HDL	0.12	0.13	-0.06	0.12

^∗^Significant correlation at the 0.05 level. ^∗∗^Significant correlation at the 0.01 level.

**Table 6:** Cut-offs, sensitivity, and specificity of lipid and blood pressure parameters.

Parameter	Youden index	Cut-off	Sensitivity	Specificity
Systolic blood pressure (mmHg)	0.31	>150	79.2	52.1
Diastolic blood pressure (mmHg)	0.14	>99	54.2	60.3
Total cholesterol (mmol/L)	0.23	>3.3	100.0	23.3
Triglyceride (mmol/L)	0.30	>0.77	66.7	63.0
HDL cholesterol (mmol/L)	0.30	≤1.7	100.0	30.1
LDL cholesterol (mmol/L)	0.34	>2.3	83.3	50.7
TC/HDL	0.34	>3.12	87.5	46.6
TG/HDL	0.26	>1.4	70.8	54.8

## Data Availability

Data are part of composite data from a larger project and could be extracted and provided if required.
